# Agr Regulation of Streptococcal Pyrogenic Exotoxin A in *Staphylococcus aureus*

**DOI:** 10.17912/micropub.biology.000795

**Published:** 2023-04-24

**Authors:** Patrick Schlievert, Samuel Kilgore, Donald Leung

**Affiliations:** 1 Microbiology and Immunology, University of Iowa; 2 Pediatrics, National Jewish Health

## Abstract

Group A streptococcal pyrogenic exotoxins (SPEs A, B, and C) are superantigens. SPE A shares high sequence similarity with *Staphylococcus aureus* enterotoxins (SEs) B and C. Since SPE A is bacteriophage-encoded, we hypothesized that its gene ( *speA* ) was acquired from *S. aureus* . *speA* , when cloned into *S. aureus* , was stably expressed, its protein resistant to proteases, and the gene under accessory gene regulator control. *speA* was acquired by streptococci from cross-species transduction. *speB* was not expressed in *S. aureus.* SPE C was degraded by staphylococcal proteases. The genes *speB* and *speC* were not recently acquired from *S. aureus.*

**Figure 1.**
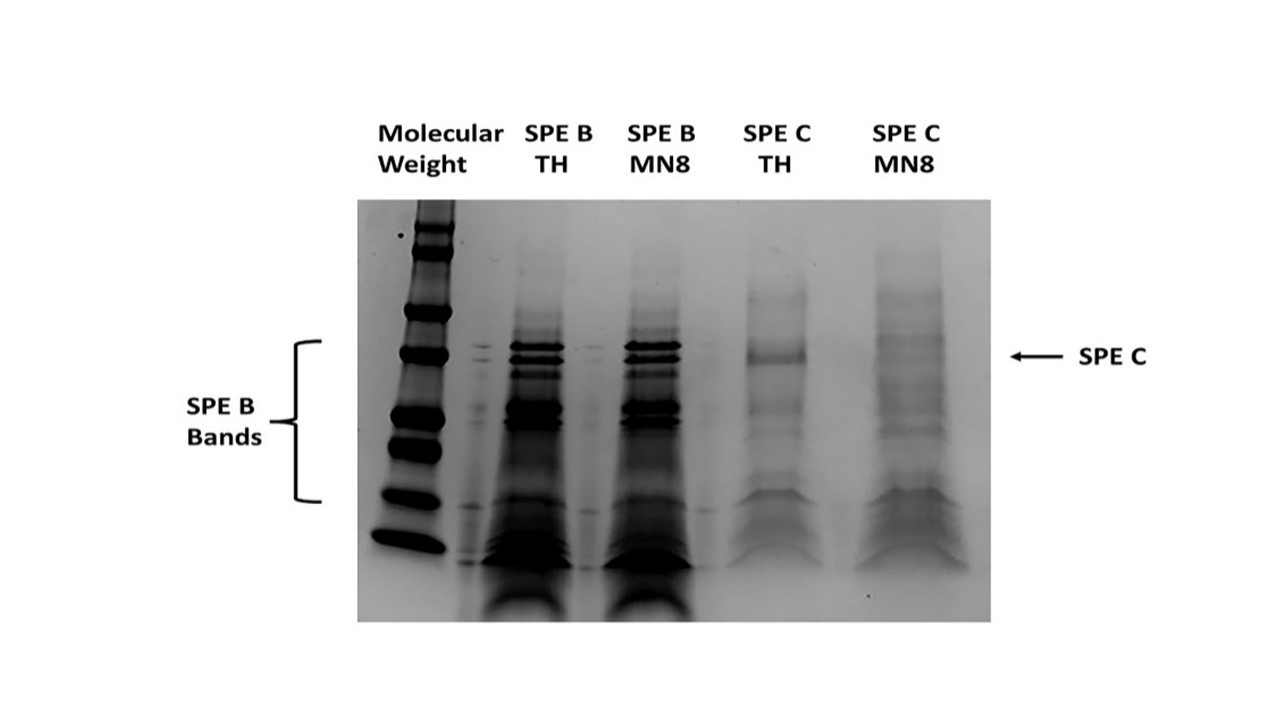
**Figure 1.** Sodium dodecyl sulfate polyacrylamide gel electrophoresis of purified SPE B and SPE C after treatment with protease-containing culture fluids of *S. aureus* MN8 (MN8) or control TH broth.

## Description

**Description**

Group A streptococcal pyrogenic exotoxins (SPEs; scarlet fever toxins) are members of the larger superantigen family of Gram-positive cocci toxins (McCormick, et al. 2001b; Spaulding, et al. 2013) . The major SPEs include SPEs A-C and F-M serotypes, with SPEs A-C being the original erythrogenic toxins described in the early to mid-1900s. All SPEs except SPE J were originally encoded by bacteriophages, though the majority are now chromosomal, based on bacteriophage mutations not allowing excision (Goshorn and Schlievert 1989; Johnson, et al. 1986b). These most often have become pathogenicity islands, trapped in the chromosomes of producing strains. The gene for SPE J is present in the chromosome of all Group A streptococcal strains and is not encoded on bacteriophages (McCormick, et al. 2001a).

SPEs are the causes of streptococcal toxic shock syndrome (STSS) primarily through the superantigen activation of the immune system; we refer to them as pyrogenic toxin superantigens (Belani, et al. 1991; Cone, et al. 1987; Lee and Schlievert 1989; Stevens, et al. 1989) . This effect is mediated by the pyrogenic toxin superantigen forming cross bridges between the alpha and/or beta chains of major histocompatibility complex (MHC) II molecules on antigen presenting cells and the variable part of the beta chain of T cell receptors (McCormick, et al. 2001b; Spaulding, et al. 2013) . This process leads to activation of up to 50% of T cells and all macrophages, with consequent downstream production of cytokines that cause fever, hypotension and shock, and the scarlet fever rash.

When our laboratory first received approval from the United States Recombinant DNA Advisory Committee (RAC) to clone and sequence the genes for SPEs A-C in the early 1980s, we showed that SPE A was highly related in nucleotide and amino acid sequence to staphylococcal enterotoxins B and C (Johnson, et al. 1986a), two superantigens that were reclassified in 2001 by the Centers for Disease Control and Prevention as select agents of bioterrorism. Prior to that time, the RAC had already given us permission to clone also the staphylococcal pyrogenic toxin superantigens, including toxic shock syndrome toxin-1 (TSST-1) and enterotoxins A-G (excluding F which later became known as TSST-1), and enterotoxin-like superantigens. With the reclassification of SEs as select agents, we destroyed all plasmids and clones encoding SEs.

As a result of showing the similarity to SEs B and C, we hypothesized that SPE A was derived from *S. aureus* by specialized transduction. The bacteriophages encoding both SPEs A and C are terminally redundant, with approximately 15% extra space at the termini to acquire 4-5 kilobases of additional DNA (Goshorn and Schlievert 1989; Johnson and Schlievert 1983) . The genes for SPEs A and C, including promoters and terminators are about 1 kilobase each.

The staphylococcal superantigens TSST-1 and enterotoxins B and C are partially under control of the global regulator of exotoxin production, termed accessory gene regulator ( *agr* ) (Recsei, et al. 1986) . We thus also proposed that if SPEs were derived from *S. aureus* , their genes and proteins would likely be stably expressed in *S. aureus* and pyrogenic toxin superantigen production under *agr* control.

We thus cloned the SPEs A, B, and C genes into a previously used vector (Kreiswirth, et al. 1987) and transformed them into an isogenic pair of *S. aureus* strains (RN4282: *agr* + and RN4256: *agr* -) (Yarwood, et al. 2002) . We then tested the ability of the resultant transformants to produce the cloned pyrogenic toxin superantigens. We observed that the SPE A gene was present in both *S. aureus* strains. SPE A was produced at 12 µg/ml by RN4282, but no SPE A production was detected from RN4256 (Table 1). These data indicate three things: 1) the SPE A gene was stable in *S. aureus* , 2) the SPE A protein was resistant to *S. aureus* proteases similar to staphylococcal enterotoxins B and C, and 3) SPE A production came under *agr* control. Thus, it is likely that SPE A and enterotoxins B and C had a shared ancestor, as was actually ruled by the RAC in the 1980s, allowing us to clone the SPE genes into *S. aureus* . We propose this ancestor was in *S. aureus* with more recent acquisition by Group A streptococci from bacteriophages.

**Table 1.** Production of streptococcal pyrogenic exotoxins by RN4282 and RN4256

**Table:** 

**Protein**	**Production in RN4282** **(µg/ml)**	**Production in RN4256** **(µg/ml)**
SPE A	12	<0.6
SPE B	<0.6	<0.6
SPE C	<0.6	<0.6

We attempted the same experiments with the SPE B and C genes (Bohach, et al. 1988; Goshorn, et al. 1988) . We were able to express both of these genes stably in *S. aureus* , but we could not detect either protein in the supernatant fluids (Table 1).

We postulated that these latter two proteins, as produced in *S. aureus* , could be degraded by proteases. This was tested by evaluating *S. aureus* strain MN8 proteases for ability to degrade SPEs B (Bohach, et al. 1988; Hauser and Schlievert 1990) and C (Goshorn, et al. 1988; Schlievert, et al. 1977) proteins. SPE B is also a cysteine protease itself and is cleaved from a pro-protease to an active protease. As we purify SPE B we typically see the pro-protease forms and various cleavage products. When incubated with *S. aureus* MN8 cultures containing proteases, there was no degradation of any of the SPE B protease bands ( Figure 1 ). In contrast, the SPE C protein was completely degraded by the MN8 culture fluids, compared to SPE C incubated with TH broth. The data from these two proteins collectively indicate: 1) The lack of production of SPEs B and C by *S. aureus* RN4282 was not the result of plasmid instability, 2) SPE B protein was stable to *S. aureus* proteases, but SPE C was degraded by the proteases, 3) there must be another unknown mechanism for lack of expression of SPE B by *S. aureus* RN4282; one possibility is that SPE B protein is cleaved inside *S. aureus* which then degrades the internal machinery required for SPE B expression, and 4) these two genes and their proteins were thus not recently acquired by Group A streptococci from *S. aureus.*

## Methods

**Bacteria and SPEs.** Group A streptococcal strains T25 _3_ cured(T12) was the original source of *speA,* the gene for SPE A (Johnson and Schlievert 1984) . Strain 86-858 was the original source of *speB* , the gene for SPE B (Bohach, et al. 1988) , and as the source of SPE B protein in this study (Barsumian, et al. 1978) . Strain T18P was the original source of *speC* , the gene for SPE C (Goshorn, et al. 1988) , and as the source of the SPE C protein in this study (Schlievert, et al. 1977) . *S. aureus* strains RN4282 and RN4256 have been described previously (Kreiswirth, et al. 1983; Peng, et al. 1988; Recsei, et al. 1986; Yarwood, et al. 2002) . All organisms were maintained as -80 °C stock cultures. For use in experiments, the bacteria were cultured in Todd Hewitt broths (Difco, Detroit, MI).

Plasmids for electroporation into RN4282 and RN4256 were constructed exactly as done for *speA* in *Bacillus subtilis* (Kreiswirth, et al. 1987) . Electroporation was accomplished with use of a prior procedure. Selection of transformants was on TH agar plates with chloramphenicol (10 µg/ml). The ability of transformants to produce SPEA, B, or C was by quantitative double immunodiffusion (Schlievert and Blomster 1983; Schlievert and Kelly 1984) . Antisera for use in double immunodiffusion was prepared by immunization (three times) every other week in rabbits with highly purified SPEs emulsified in Freund’s incomplete adjuvant. The rabbits were bled one week after the last injection, and serum collected after clotting. The reactivity of the antisera was such that SPEs could be detected at 0.6 µg/ml. Purified SPEs were prepared by microbial culture to stationary phase, and combinations of ethanol precipitation and thin-layer isoelectric focusing (Schlievert, et al. 1977) .

**Protease assay.** Purified SPE B and C were treated 48 h with *S. aureus* strain MN8 sterile culture fluids after stationary phase growth in TH broth with shaking (200 revolutions/min) at 37 °C, or uninoculated TH broth. MN8 is a menstrual toxic shock syndrome strain of *S. aureus* (Schlievert and Kelly 1984) that produces at least six different proteases, including cysteine proteases and serine proteases. Subsequently, the preparations were subjected to sodium dodecyl sulfate polyacrylamide gel electrophoresis with Coomassie brilliant blue staining of gels.
